# Polygenic Score for Body Mass Index Is Associated with Weight Loss and Lipid Outcomes After Metabolic and Bariatric Surgery

**DOI:** 10.3390/ijms26157337

**Published:** 2025-07-29

**Authors:** Luana Aldegheri, Chiara Cipullo, Natalia Rosso, Eulalia Catamo, Biagio Casagranda, Pablo Giraudi, Nicolò de Manzini, Silvia Palmisano, Antonietta Robino

**Affiliations:** 1 Institute for Maternal and Child Health—IRCCS Burlo Garofolo, 34137 Trieste, Italy; luana.aldegheri@burlo.trieste.it (L.A.); chiara.cipullo@burlo.trieste.it (C.C.); eulalia.catamo@burlo.trieste.it (E.C.); 2 Metabolic Liver Disease Unit, Fondazione Italiana Fegato—ONLUS, Area Science Park, Basovizza, 34149 Trieste, Italy; natalia.rosso@fegato.it (N.R.); pablo.giraudi@fegato.it (P.G.); 3 Surgical Clinic Division, Cattinara Hospital, Azienda Sanitaria Universitaria Giuliano Isontina, 34149 Trieste, Italy; biagio.casagranda@asugi.sanita.fvg.it (B.C.); demanzini@units.it (N.d.M.); silvia.palmisano@asugi.sanita.fvg.it (S.P.); 4 Department of Medical, Surgical and Health Sciences, University of Trieste, 34127 Trieste, Italy

**Keywords:** obesity, metabolic and bariatric surgery, polygenic score

## Abstract

Metabolic and bariatric surgery (MBS) is an effective treatment for severe obesity, though individual responses vary widely, partly due to genetic predisposition. This study investigates the association of a body mass index (BMI) polygenic score (PGS) with weight loss and metabolic outcomes following surgery. A cohort of 225 patients undergoing MBS was analyzed at baseline (T_0_), six (T_6_), and twelve (T_12_) months, with anthropometric and biochemical parameters recorded at each time point. Total weight loss (TWL) and excess weight loss (EWL) percentages were calculated. PGS was computed using the LDpred-grid Bayesian method. The mean age was 45.9 ± 9.4 years. Males had a higher baseline prevalence of type 2 diabetes (T2D) and comorbidities (*p* < 0.001). Linear regression analysis confirmed an association between PGS and baseline BMI (*p* = 0.012). Moreover, mediation analysis revealed that baseline BMI mediated the effect of the PGS on %TWL at T_12_, with an indirect effect (*p*-value = 0.018). In contrast, high-density lipoprotein-cholesterol (HDL-C) at T_6_ and triglycerides (TG) at T_12_ showed direct associations with the PGS (*p*-value = 0.004 and *p*-value = 0.08, respectively), with no significant mediation by BMI. This study showed a BMI-mediated association of PGS with %TWL and a direct association with lipid changes, suggesting its potential integration into personalized obesity treatment.

## 1. Introduction

Obesity is a multifactorial disease influenced by a complex interplay of behavioral, psychosocial, environmental factors and genetic predisposition [1]. According to the World Health Organization (WHO), in 2022, one in eight people worldwide were obese, and 2.5 billion adults (≥18 years) were overweight. Moreover, by 2024, 35 million children under five years of age were classified as overweight, highlighting the early onset and widespread nature of this condition [2].

Of all available treatment options, metabolic and bariatric surgery (MBS) is currently the most effective intervention for severe obesity, leading to substantial and sustained weight loss, as well as significant improvements in obesity-related comorbidities, such as type 2 diabetes (T2D), cardiovascular disease, obstructive sleep apnea, dyslipidemia, and hypertension [3,4,5].

Among the surgical techniques, sleeve gastrectomy (SG) and Roux-en-Y gastric bypass (RYGB) represent the two most performed bariatric procedures, each inducing substantial weight loss through different mechanisms. SG reduces stomach volume, alters gut hormones, such as ghrelin and peptide YY, and changes gastric motility [6]. Conversely, RYGB involves the creation of a small gastric pouch and rerouting the small intestine to this pouch, thereby bypassing much of the stomach and duodenum [7].

Although RYGB is considered the gold standard after restrictive surgery, one anastomosis gastric bypass (OAGB), also known as a mini gastric bypass, is a viable alternative due to its technical simplicity, reversibility, and shorter operating time [8]. The procedure involves suturing the stomach from its lower part to create a long gastric pouch; the rest of the stomach remains inside, but food does not enter [9]. While OAGB involves creating a single connection between the stomach and the small intestine, RYGB involves an additional connection between two sections of the small intestine [10].

Despite the overall success of these procedures, post-operative weight loss outcomes vary widely among individuals [11,12]. This inter-individual variability represents a significant clinical challenge and underscores the need to improve our knowledge of the factors that influence surgical efficacy. Understanding these determinants is essential to optimize patient selection and post-operative management.

Genetic factors have increasingly been recognized as important contributors to both obesity susceptibility and response to weight loss interventions [13]. Inherited predisposition to obesity can result from both monogenic and polygenic mechanisms. In rare cases, obesity may be the result of single-gene mutations that exert large effects on energy balance or adipose tissue regulation. However, for most individuals, no single causative mutation has been identified and genetic susceptibility is explained by the combined influence of numerous genetic variants, each exerting a modest effect on body weight regulation [14]. Indeed, in recent years, polygenic scores (PGSs), which combine the effects of numerous genetic variants carried by an individual associated with a given trait or disease, have emerged as promising tools for quantifying inherited predisposition to obesity [15,16].

Several studies have demonstrated that individuals with a higher PGS for body mass index (BMI) or obesity are more likely to gain weight over time and have a greater risk of developing obesity-related diseases [17,18]. However, the role of polygenic risk in predicting weight loss following MBS remains an area of active investigation. While some evidence suggests that a greater genetic predisposition to obesity may be associated with reduced post-surgical weight loss or greater weight regain over time after surgery [19,20,21], other studies report no significant association [22]. These inconsistencies may arise from differences in PGS construction, sample sizes, ethnic backgrounds of study populations, or types of surgical procedure analyzed.

Given the growing interest in precision medicine approaches to metabolic surgery, clarifying the relationship between obesity-related polygenic risk and weight loss trajectories after MBS is of both clinical and scientific importance. Therefore, this study aims to assess whether a BMI-based PGS is associated with weight loss outcomes following bariatric surgery, measured by total weight loss (TWL). Moreover, the association of PGS with other metabolic outcomes, such as lipid profile and hepatic enzyme levels, was evaluated to better understand the broader physiological impact of genetic risk in the context of metabolic surgery.

## 2. Results

### 2.1. Sample Characteristics

The study cohort, composed mainly of females (73.8%), had a mean age of 45.9 ± 9.4, ranging from 20 to 63. Males showed a higher percentage of T2D at T_0_ and other comorbidities compared to females (*p*-value < 0.001) (Table 1).

High-density lipoprotein-cholesterol (HDL-C), low-density lipoprotein-cholesterol (LDL-C), and total cholesterol (TC) are significantly higher in females at all time points, while males had higher levels of triglycerides (TG) at T_0_ (*p*-value = 0.013) (Table 2).

Regarding hepatic profile, alanine aminotransferase (ALT), aspartate aminotransferase (AST) and gamma-glutamyl transferase (GGT) were significantly different between males and females at T_0_ (*p*-value < 0.001). Differences in hepatic enzymes were also found for AST at T_6_ (*p*-value = 0.018) and T_12_ (*p*-value = 0.007) and for GGT at T_6_ (*p*-value < 0.001) and T_12_ (*p*-value < 0.001) (Table 2).

Compared to RYGB and OAGB patients, those who underwent SG had a significantly higher BMI at T_0_ (*p*-value_RYGB_ = 0.006, *p*-value_OAGB_ < 0.001), at T_6_ (*p*-value_RYGB_ = 0.006, *p*-value_OAGB_ < 0.001), and at T_12_ (*p*-value_RYGB_ < 0.001, *p*-value_OAGB_ < 0.001), while no differences were found when comparing RYGB and OAGB patients with each other (Figure S1). Obese subjects who underwent SG had a significant lower %TWL compared to those who underwent RYGB at T_6_ (*p*-value = 0.012), but not at T_12_ (Figure S2).

### 2.2. Association of PGS with Weight Loss and Metabolic Outcomes

Linear regression analysis with age and sex as covariates confirmed in our cohort that PGS is associated with BMI at T_0_ (*p*-value = 0.012, beta = −4.7, R2 = 5%).

Therefore, to study the effect of PGS on weight loss and metabolic outcomes, mediation analysis where BMI at T_0_ was considered as the mediator was conducted. This analysis showed that baseline BMI significantly mediated the association between the PGS and post-operative outcomes (Table 3). Specifically, for %TWL at T_12_, the indirect effect was significant (*p*-value = 0.018), while the direct effect did not reach statistical significance (*p*-value = 0.144). The total effect was statistically significant (*p*-value = 0.028), and approximately 39% of the total effect was mediated by baseline BMI (*p*-value = 0.046). Similarly, for %TWL at T_6_, the indirect effect was also significant (*p*-value = 0.014), but neither the direct nor the total effect reached statistical significance.

Regarding %EWL at T_6_ and T_12_, the average casual mediation effect (ACME) was significant (*p*-value = 0.024 and *p*-value = 0.012, respectively), whereas the direct and total effects were not statistically significant.

Mediation analysis for lipids indicated a significant total effect of the PGS on variation of HDL-C at T_6_ (*p*-value < 0.001) and TG at T_12_ (*p*-value = 0.026) post-surgery. The direct effect was significant for both HDL-C at T_6_ (*p*-value = 0.004) and TG at T_12_ (*p*-value = 0.008), while the indirect effect mediated by baseline BMI did not reach statistical significance.

The proportion mediated was not significant, suggesting that the effect of the PGS on triglyceride changes is primarily direct, rather than mediated by baseline BMI (Table 4).

## 3. Discussion

This study investigated the role of a PGS for BMI in influencing weight loss and metabolic outcomes following MBS.

Our findings show that genetic predisposition to higher BMI, as captured by the PGS, has a significant indirect influence on weight loss (measured as %TWL) at twelve months after surgery, with baseline BMI acting as a key mediator. Specifically, nearly 40% of the effect of the PGS on %TWL was mediated by baseline BMI, highlighting the role of genetic predisposition in shaping pre-operative body composition, which, in turn, influences the magnitude and variability of weight loss in response to MBS.

In this study, we also identified a direct association of the PGS with changes in lipid profile after bariatric surgery, specifically changes in HDL-C at six months and TG levels at twelve months postoperatively. Unlike %TWL, these associations with lipids were not mediated by baseline BMI, suggesting that genetic predisposition to obesity may influence the metabolic response to surgery, particularly with respect to lipid metabolism. This aligns with the concept of “metabolic responders” versus “non-responders” to MBS, suggesting that genomic factors may contribute to such variability [23].

In fact, MBS is increasingly recognized not only as a weight reduction strategy, but as a metabolic intervention with far-reaching effects on cardiovascular and endocrine health. Accordingly, current definitions of surgical success now emphasize improvements in metabolic outcomes, such as glycemic control, lipid levels, and cardiometabolic risk reduction, rather than weight loss alone [24,25,26]. Lipid profile changes, including increases in HDL-C and reductions in TG, are among the most clinically meaningful metabolic shifts observed after surgery and are strong predictors of cardiovascular risk reduction [27,28].

Moreover, our findings agree with prior research indicating shared genetic determinants between obesity and lipid traits [29,30].

The results of this study are also consistent with previous literature highlighting sex-specific metabolic responses and support the need for gender-tailored clinical management in patients with obesity [31,32]. In our cohort, sex differences emerged in several clinical variables: males exhibited both higher TG levels and prevalence of T2D and other comorbidities at baseline, as well as elevated hepatic enzyme levels throughout follow-up, whereas females consistently presented higher levels of HDL-C, LDL-C, and TC. Moreover, regarding the type of surgery, our study showed that patients who underwent SG had a significantly higher BMI at all time points compared to those who underwent RYGB or OAGB and showed a lower %TWL at six months after surgery compared to RYGB patients, confirming previous studies that have already reported that the type of bariatric surgery influences the weight loss trajectory [33,34].

The use of mediation analysis to investigate the complex interplay between genetic predisposition, baseline BMI, and both anthropometric and metabolic outcomes following bariatric surgery is a strength of this study. This method allowed us to distinguish direct from indirect genetic effects and to uncover the mediating role of pre-operative BMI in influencing weight loss outcomes.

This study has limitations, however. The sample size, while adequate for initial exploration, may not capture the full spectrum of genetic variability, and replication in larger, independent cohorts is necessary. Furthermore, the observed effect of PGS on weight loss outcomes was modest in magnitude and largely mediated by baseline BMI. However, given the polygenic architecture of obesity and the multifactorial nature of post-surgical weight loss, this result is expected, suggesting that environmental and behavioral factors likely play a substantial role and should be integrated into future models. Importantly, our results highlight that BMI-PGS should not be considered a replacement for established clinical predictors, such as baseline BMI, but rather a complementary source of information that may improve risk stratification or identify individuals who may benefit from more intensive follow-up.

Therefore, as genotyping becomes more accessible in clinical settings, although PGS cannot yet inform individual clinical decisions, integrating genetic data into clinical decision-making, alongside demographic, clinical, and behavioral factors, could enhance personalized approaches to obesity treatment, ultimately improving patient outcomes after bariatric surgery.

## 4. Materials and Methods

### 4.1. Participants

A total of 225 severely obese subjects undergoing MBS were enrolled and followed up for one year. The inclusion criteria were: well-informed and motivated patients with acceptable surgical risk; failure of previous non-surgical weight loss treatments; aged between 18 and 65 years old; and a BMI (body mass index, kg/m^2^) > 40 kg/m^2^ or >35 kg/m^2^, with comorbidities related to obesity. Surgical interventions consisted of RYGB (39.1%), OAGB (27.1%), and SG (33.8%). Patients were evaluated at three time points: before surgery (T_0_) and at six (T_6_) and twelve (T_12_) months after surgery. All subjects gave their written informed consent before participating in this study, approved by protocol N. 16,637 Local Ethical Committee (Comitato Etico Unico Regionale FVG, Udine, Italy).

### 4.2. Data

Demographic and clinical data were collected for each participant at all three time points. Weight (W) and height were taken on a digital weighing scale, and BMI was calculated (kg/m^2^).

The total weight loss (%TWL) at T_6_ and T_12_ was calculated as (Weight loss/WT_0_) × 100.

The excess weight loss (%EWL) at T_6_ and T_12_ was calculated as (Weight loss/Excess Weight) × 100 (where excess weight is T_0_ weight − ideal weight).

Data on diabetes diagnosis, ongoing diabetic therapy, and the presence of other comorbidities were also recorded. Moreover, lipid profile (HDL-C, LDL-C, TC and TG), hepatic enzymes (ALT, AST and GGT), and HbA1c were measured.

Delta values for HDL-C, LDL-C, TC, TG, ALT, AST, GGT, and HbA1c were calculated as the difference between the variable at T_6_ and T_0_ for ΔT_6_ and between the variable at T_12_ and T_0_ for ΔT_12_.

### 4.3. DNA Extraction and Genotyping

For each participant, 23 ± 9 mg of liver tissue was collected and stored at −80 °C until DNA extraction, which was performed with the Quick-DNA/RNA^TM^ Miniprep Plus Kit (Zymo Research, Irvine, CA, USA), following the manufacturer’s protocol. Frozen samples were homogenized in 300 μL of DNA/RNA Shield™ (1X) using a bead homogenizer (Bead Ruptor, Omni Inc, Kennesaw, GA, USA), then incubated with 15 μL of Proteinase K and 30 μL of PK Digestion Buffer at room temperature (RT) for 30 min. DNA/RNA Lysis Buffer was added in a 1:1 ratio, and the lysates were processed through Spin-Away™ Filters and purification columns, following the protocol’s instruction (Zymo Research, Irvine, CA, USA). DNA was then eluted in 50 μL of DNase/RNase-free water. Subsequently, the concentration (ng/µL) of the nucleic acid was determined using the NanoDrop^®^ 1000 spectrophotometer (Thermo Fisher Scientific, Waltham, MA, USA). The absorbance ratios at 260/280 and 260/230 were evaluated as indicators of the DNA purity. Then, 96-well plates were prepared with DNA samples adjusted to a final concentration of 65 ng/μL in a total volume of 10 μL per well. Genotyping was conducted by Illumina Infinium Global Screening Array (GSA v3.0).

### 4.4. Quality Control, Imputation, and PGS Calculation

Quality control (QC) was performed on genotyped data to remove samples with low genotype calls (<95%), heterozygosity with six standard deviations (SDs) away from the mean, identity by descent (IBD) proportion > 0.4, and to remove duplicate single nucleotide polymorphisms (SNPs) and those with missing call rate > 1% or with a Hardy–Weinberg equilibrium (HWE) *p*-value < 1 × 10^−6^. Moreover, the PLINKv1.9—check-sex function made it possible to assess the potential sex discrepancies between the predicted and reported sex of individuals [35,36]. Samples with a non-European ancestry detected by principal component analysis were excluded. After the QC procedure, the genotyped data were subjected to a genotyping imputation using standard procedures. Briefly, genotype phasing was performed with the software Eagle2 V2.4.1 [37], while impute5 V1.1.5 software [38] and IGRP panel [39] were used for imputation. Variants with imputation scores < 0.8 were removed.

The dedicated LDpred2 program using a Bayesian approach was used to calculated PGS [40]. Imputed data as target data and summary statistics from a published BMI Genome-Wide Association Study (GWAS) [41] as base data were used. SNPs from the BMI GWAS were matched with SNPs from our imputed data and only SNPs that were present in both were considered (*n* = 871,416). For each SNP, the number of the alleles (0, 1, or 2) associated with BMI was multiplied by the effect estimated in the GWAS. The PGS for each individual was an average of the weighted BMI-associated alleles.

### 4.5. Statistical Analyses

Continuous variables are described as mean ± SD when normally distributed, and as median (IQR) when not normally distributed. Categorical variables are reported as percentages. Differences between male and female were assessed using the *t*-test or Wilcoxon rank sum test for continuous variables that were normally or not normally distributed, respectively; while the Chi-squared test was used for categorical variables. Due to their not normal distributions, lipids and hepatic enzymes data were log-transformed prior to calculating the Δ values at T_6_ and T_12_. To study the effect of PGS on post-surgical weight loss and metabolic outcomes, we conducted a causal mediation analysis using the mediate() function from the R mediation package (V4.4.2). The average causal mediation effect (ACME), average direct effect (ADE), total effect, and proportion mediated were estimated using nonparametric bootstrapping with 1000 simulations. The PGS was treated as the exposure, baseline BMI as the mediator, and post-operative weight loss and metabolic outcomes as the dependent variables. The analyses were adjusted for age, sex, and type of surgery.

Linear regression was conducted to test the association between PGS and baseline BMI, with correction for age and sex. ANOVA and the post-hoc Tukey’s Honest Significant Difference (HSD) test were used to study the relationship between %TWL and type of surgery and between BMI and type of surgery. All statistical analyses were conducted in RStudio (V4.4.2) [42].

## Figures and Tables

**Table 1:** Sample characteristics at enrollment by gender. *p*-value from Wilcoxon test and Chi-squared test for continuous and categorical variables, respectively. RYGB, Roux-en-Y gastric bypass; OAGB, one anastomosis gastric bypass; SG, sleeve gastrectomy.

Characteristics	All (*n* = 225)	Males (*n* = 59)	Females (*n* = 166)	*p*-Value
Age (mean ± SD)	45.9 ± 9.4	47.0 ± 9.1	45.5 ± 9.5	0.24
Surgery (%) RYGB OAGB SG	39.1 27.1 33.8	36.0 25.0 39.0	40.4 27.7 31.9	0.61
Diabetes (% Yes)	27.6	52.5	18.7	<0.001
Comorbidities (% Yes)	66.2	84.7	59.6	<0.001

**Table 2:** Anthropometric, lipids, and hepatic enzymes at T_0_, T_6_, and T_12_. *p*-value from *t*-test or Wilcoxon test for normally and not normally distributed data, respectively. BMI, body mass index; %TWL, percentage of total weight loss; % EWL, percentage of excess weight loss; IQR, interquartile range; HDL-C, high-density lipoprotein-cholesterol; LDL-C, low-density lipoprotein-cholesterol; TC, total cholesterol; TG, triglycerides; ALT, alanine aminotransferase; AST, aspartate aminotransferase; GGT, gamma-glutamyl transferase; HbA1c, glycated hemoglobin.

Characteristics	All	Males	Females	*p*-Value
BMI, kg/m^2^, mean ± SD				
T_0_	42.8 ± 5.5	43.6 ± 5.5	42.5 ± 5.5	0.19
T_6_	31.5 ± 4.3	32 ± 4.3	31.3 ± 4.3	0.33
T_12_	28.8 ± 4.4	29.4 ± 3.6	28.5 ± 4.6	0.13
TWL, %, mean ± SD				
T_6_	26.2 ± 6.2	26.3 ± 5.6	26.1 ± 6.3	0.45
T_12_	32.4 ± 8.5	31.7 ± 8.1	32.6 ± 8.7	0.49
EWL, %, mean ± SD				
T_6_	66.2 ± 18.5	65.1 ± 19.0	66.6 ± 18.3	0.47
T_12_	81.7 ± 23.1	77.7 ± 20.0	83.0 ± 24.1	0.46
HDL-C, mg/dL, median (IQR)				
T_0_	48 (15)	40 (11.5)	50 (13)	<0.001
T_6_	50 (17)	44 (14)	53 (16)	0.002
T_12_	57 (17)	50.5 (18.5)	60 (16.2)	<0.001
LDL-C, mg/dL, median (IQR)				
T_0_	133 (43.6)	114.4 (53)	140.2 (41.3)	<0.001
T_6_	102.2 (42)	92.6 (45.9)	104 (38.3)	0.03
T_12_	98.6 (47.9)	84.4 (47)	101.2 (45)	0.002
TC, mg/dL, median (IQR)				
T_0_	209 (60)	180 (60)	215 (51)	<0.001
T_6_	174 (49.5)	147 (56)	178.5 (43)	0.003
T_12_	175 (52.5)	151 (50)	181 (48)	<0.001
TG, mg/dL, median (IQR)				
T_0_	117 (66)	151 (104.5)	113 (55.5)	0.013
T_6_	91 (39)	87 (50.5)	93 (35.8)	0.48
T_12_	83 (39.5)	79 (34)	84 (41)	0.83
ALT, U/L, median (IQR)				
T_0_	24 (17)	32 (23)	21 (13.8)	<0.001
T_6_	18 (10)	21 (16)	17 (8)	0.08
T_12_	17 (13.3)	21 (17.5)	16 (12)	0.07
AST, U/L, median (IQR)				
T_0_	21 (10)	26 (14.5)	20 (8)	<0.001
T_6_	19 (8)	21 (7.8)	18 (7)	0.018
T_12_	19 (9)	22 (10)	18 (7)	0.007
GGT, U/L, median (IQR)				
T_0_	24 (25)	39 (33)	21.5 (17.8)	<0.001
T_6_	15 (12.8)	21.5 (18.8)	12 (11)	<0.001
T_12_	15 (11.5)	21 (20)	13 (8.8)	<0.001
HbA1c, %, median (IQR)				
T_0_	5.8 (0.7)	6.2 (1.2)	5.8 (0.5)	0.014
T_6_	5.4 (0.6)	5.4 (0.6)	5.4 (0.6)	0.90
T_12_	5.3 (0.6)	5.2 (0.7)	5.4 (0.5)	0.24

**Table 3:** Results of mediation analysis for weigh loss outcomes. Sex, age, and type of surgery have been used as covariates. %TWL, percentage of total weight loss; % EWL, percentage of excess weight loss. ACME, Average Causal Mediation Effect; ADE, Average Direct Effect; CI, confidence interval.

Variable	Estimate	*p*-Value	95% CI Lower	95% CI Upper
%TWL T_6_				
ACME	−1.22	0.014	−2.56	−0.19
ADE	−1.07	0.542	−4.97	3.03
Total effect	−2.29	0.278	−6.23	1.76
Proportion mediated	0.53	0.292	−3.61	3.50
%TWL T_12_				
ACME	−2.62	0.018	−5.08	−0.53
ADE	−4.06	0.144	−9.70	1.43
Total effect	−6.68	0.028	−12.47	−1.09
Proportion mediated	0.39	0.046	0.030	1.48
%EWL T_6_				
ACME	7.95	0.024	1.27	14.58
ADE	−3.73	0.482	−13.62	6.42
Total effect	4.22	0.524	−7.65	16.31
Proportion mediated	1.88	0.512	−18.15	11.77
%EWL T_12_				
ACME	7.31	0.012	1.55	14.47
ADE	−10.94	0.170	−24.26	4.75
Total effect	−3.63	0.674	−18.12	12.67
Proportion mediated	−2.01	0.686	−9.95	10.85

**Table 4:** Results of mediation analysis for lipids Δ values. Sex, age, and type of surgery have been used as covariates. Δ values for HDL-C, LDL-C, TG and TC were obtained as the difference between the values at T_6_ and T_0_ for ΔT_6_ and the difference between the values at T_12_ and T_0_ for ΔT_12_. HDL-C, high-density lipoprotein-cholesterol; LDL-C, low-density lipoprotein-cholesterol; TG, triglycerides; TC, total cholesterol; ACME, Average Causal Mediation Effect; ADE, Average Direct Effect, CI; confidence interval.

Variable	Estimate	*p*-Value	95% CI Lower	95% CI Upper
ΔHDL-C T_6_				
ACME	0.00007	0.95	−0.046	0.04
ADE	0.3	0.004	0.062	0.6
Total effect	0.3	<0.001	0.084	0.6
Proportion mediated	0.0002	0.95	−0.1	0.3
ΔHDL-C T_12_				
ACME	−0.02	0.34	−0.08	0.015
ADE	0.17	0.12	−0.03	0.46
Total effect	0.15	0.14	−0.04	0.41
Proportion mediated	−0.13	0.4	−0.8	0.78
ΔLDL-C T_6_				
ACME	−0.01	0.78	−0.096	0.037
ADE	0.4	0.076	−0.029	0.998
Total effect	0.4	0.058	−0.007	0.95
Proportion mediated	−0.028	0.73	−0.28	0.34
ΔLDL-C T_12_				
ACME	0.024	0.28	−0.016	0.08
ADE	−0.073	0.59	−0.32	0.18
Total effect	−0.048	0.73	−0.29	0.2
Proportion mediated	−0.5	0.82	−3.54	2.5
ΔTG T_6_				
ACME	−0.003	0.89	−0.057	0.048
ADE	−0.19	0.21	−0.47	0.11
Total effect	−0.2	0.18	−0.47	0.09
Proportion mediated	0.016	0.98	−1.1	0.9
ΔTG T_12_				
ACME	0.056	0.082	−0.0056	0.15
ADE	−0.34	0.008	−0.6	−0.096
Total effect	−0.3	0.026	−0.55	−0.04
Proportion mediated	−0.18	0.108	−1.15	0.034
ΔTC T_6_				
ACME	0.008	0.54	−0.02	0.04
ADE	0.068	0.48	−0.09	0.25
Total effect	0.075	0.37	−0.08	0.25
Proportion mediated	0.1	0.8	−1.7	1.88
ΔTC T_12_				
ACME	−0.004	0.90	−0.063	0.038
ADE	0.025	0.78	−0.16	0.25
Total effect	0.02	0.79	−0.16	0.21
Proportion mediated	−0.2	0.81	−3.3	2.75

No significant associations with hepatic enzymes (ALT, AST, and GGT) and HbA1c (glycated hemoglobin) have emerged (Table S1).

## Data Availability

The raw data supporting the conclusions of this article will be made available by the authors upon request.

## Supplementary Materials

The following supporting information can be downloaded at: https://www.mdpi.com/article/10.3390/ijms26157337/s1.
