# Supplementary material for: Polygenic Score for Body Mass Index Is Associated with Weight Loss and Lipid Outcomes After Metabolic and Bariatric Surgery
Source: Int J Mol Sci. 2025 Jul 29;26(15):7337. doi: 10.3390/ijms26157337 (PMC12347326; doi:10.3390/ijms26157337)
Supplement: Supplementary file 1 [file ijms-26-07337-s001.zip › ijms-3709088-supplementary.pdf]

Supplementary Materials

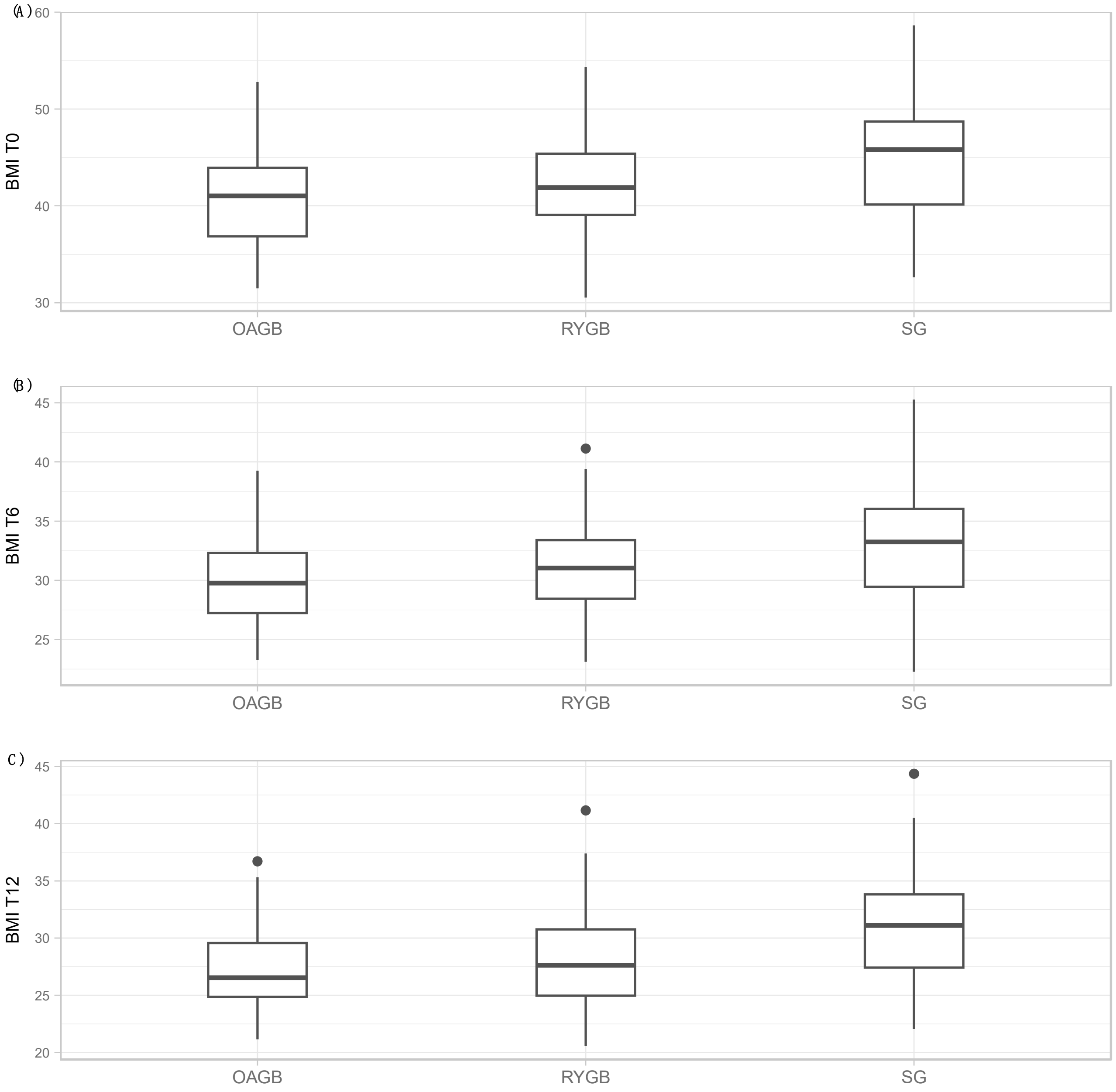

Supplementary Figure S1. Distribution of body mass index (BMI) at T_0 (A), T_6 (B) and T_{12} (C) by type of surgery (OAGB, RYGB, SG). BMI, body mass index; OAGB, one anastomosis gastric bypass; RYGB, Roux-en-Y Gastric bypass; SG, sleeve gastrectomy.

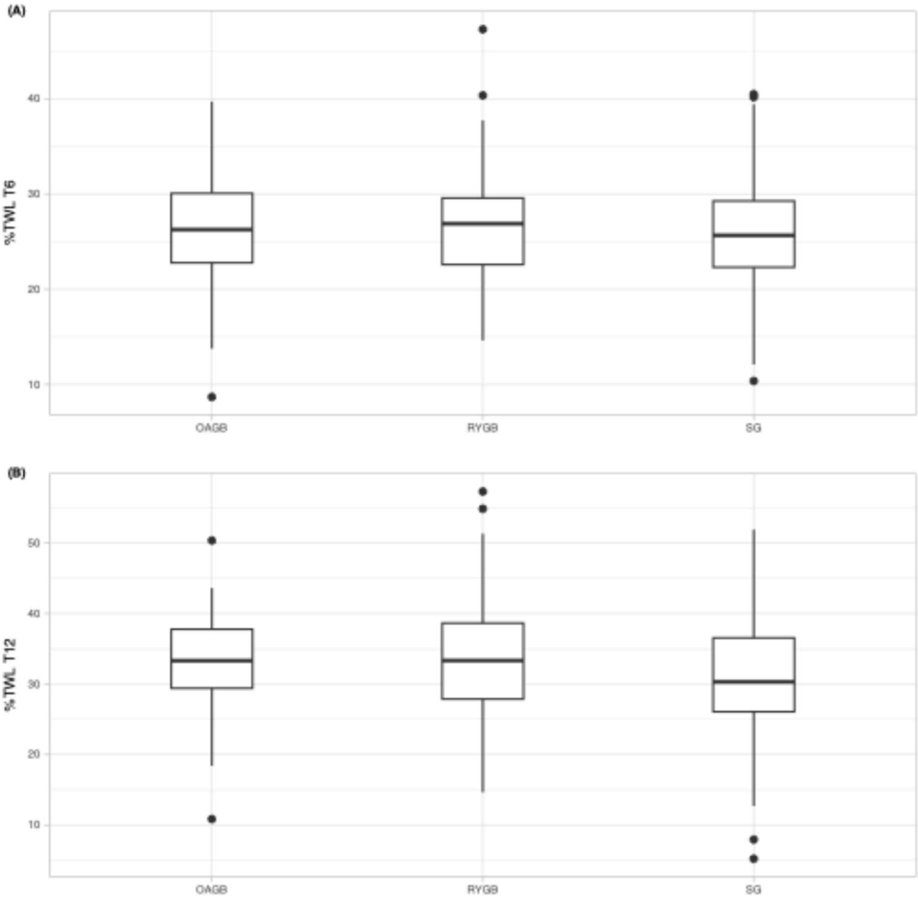

Supplementary Figure S2. Distribution of percentage of total weight loss (%TWL) at T_6 (A) and T_{12} (B) by type of surgery (OAGB, RYGB, SG). %TWL, percentage of total weight loss; OAGB, one anastomosis gastric bypass; RYGB, Roux-en-Y Gastric bypass; SG, sleeve gastrectomy.

Supplementary Table S1. Results of mediation analysis for hepatic enzymes and HbA1c Δ values. Sex, age and type of surgery have been used as covariates. Δ values for ALT, AST, GGT and HbA1c were obtained as the difference between the values at T_6 and T_0 for ΔT_6 and the difference between the values at T_{12} and T_0 for ΔT_{12}. ALT, alanine aminotransferase; AST, aspartate aminotransferase; GGT, gamma-glutamyl transferase; HbA1c, glycated hemoglobin; ACME, Average Causal Mediation Effect; ADE, Average Direct Effect, CI, confidence interval.

Variable	Estimate	p-value	95% CI Lower	95% CI Upper
**ΔALT T_6**				
ACME	0.1	0.06	0.01	0.22
ADE	-0.3	0.12	-0.67	0.09
Total effect	-0.2	0.28	-0.56	-0.17
Proportion mediated	-0.51	0.29	-4.2	3.9
**ΔALT T_{12}**				
ACME	0.14	0.20	0.027	0.31
ADE	-0.19	0.31	-0.6	0.18
Total effect	-0.05	0.75	-0.48	0.31
Proportion mediated	-3.1	0.76	-14.3	9.18
**ΔAST T_6**				
ACME	0.07	0.08	0.004	0.16
ADE	-0.12	0.37	-0.38	0.16
Total effect	-0.05	0.67	-0.3	0.21
Proportion mediated	-1.3	0.68	-7.3	10.1
**ΔAST T_{12}**				
ACME	0.1	0.20	0.009	0.21
ADE	0.06	0.746	-0.27	0.36
Total effect	0.16	0.35	-0.19	0.45
Proportion mediated	0.63	0.35	-4.9	4.7
**ΔGGT T_6**				
ACME	0.046	0.25	-0.03	0.15
ADE	-0.26	0.37	-0.8	0.31
Total effect	-0.21	0.46	-0.75	0.34
Proportion mediated	-0.22	0.59	-2.38	1.77
**ΔGGT T_{12}**				
ACME	0.06	0.18	-0.02	0.19
ADE	-0.28	0.27	-0.75	0.2
Total effect	-0.22	0.37	-0.7	0.26
Proportion mediated	-0.27	0.49	-2.2	1.9
**ΔHbA1c T_6**				
ACME	0.003	0.73	-0.014	0.028
ADE	-0.02	0.54	-0.1	0.055
Total effect	-0.02	0.59	-0.1	0.06
Proportion mediated	-0.15	0.93	-2.35	2.7
**ΔHbA1c T_{12}**				
ACME	0.02	0.07	-0.001	0.05
ADE	-0.03	0.41	-0.11	0.05
Total effect	-0.008	0.83	-0.09	0.08
Proportion mediated	-2.6	0.86	-6.4	10.5
